# Effect of applying nursing-based cognitive defusion techniques on mindful awareness, cognitive fusion, and believability of delusions among clients with schizophrenia: a randomized control trial

**DOI:** 10.3389/fpsyt.2024.1369160

**Published:** 2024-04-26

**Authors:** Ayman Mohamed El-Ashry, Eman Sameh Abd Elhay, Samah Mohamed Taha, Mahmoud Abdelwahab Khedr, Feby Saad Attalla Mansour, Amany Anwar Saeed Alabdullah, Sally Mohammed Farghaly Abdelaliem, Mona Metwally El-Sayed

**Affiliations:** ^1^ Department of Psychiatric and Mental Health Nursing, Faculty of Nursing, Alexandria University, Alexandria, Egypt; ^2^ Faculty of Nursing, Mansoura University, Mansoura, Egypt; ^3^ Department of Nursing, College of Applied Medical Sciences, Hafr Albatin University, Hafr Albatin, Saudi Arabia; ^4^ Department of Psychology, Faculty of Arts, Alexandria University, Alexandria, Egypt; ^5^ Department of Maternity and Child Health Nursing, College of Nursing, Princess Nourah bint Abdulrahman University, Riyadh, Saudi Arabia; ^6^ Department of Nursing Management and Education, College of Nursing, Princess Nourah bint Abdulrahman University, Riyadh, Saudi Arabia

**Keywords:** delusions, cognitive defusion, fusion, mindful, schizophrenia

## Abstract

**Background:**

Applying cognitive defusion techniques to enduring psychotic symptoms, such as delusions, presents both a challenge and a promising opportunity for psychiatric nurses to manage delusions among schizophrenia clients.

**Objective:**

This study aimed to examine the impact of cognitive defusion techniques on psychological flexibility, mindful awareness, cognitive fusion, and the believability of delusions in schizophrenia clients.

**Methodology:**

This study used a single-blind, parallel-arm Randomized Controlled Trial design. Over five weeks, 70 clients with schizophrenia were randomized to either the cognitive defusion intervention group (n = 35) or the control group (n = 35).

**Findings:**

The participants showed significant reductions in the believability of delusions, cognitive fusion, and psychological inflexibility immediately after the intervention and at follow-up. Notable enhancements were observed in cognitive defusion and mindfulness awareness abilities.

**Conclusion:**

Cognitive defusion techniques positively affect schizophrenia clients who struggle with persistent delusional beliefs. This underscores the importance of further investigating this approach to decrease the intensity of delusions as part of a comprehensive therapeutic intervention. Psychiatric nurses must receive training in “cognitive defusion skills” to aid schizophrenia clients in becoming more aware of their emotions and modifying their coping strategies for delusional beliefs. On August 3, 2023, the research was retrospectively registered under the reference number NCT05759091 as a randomized clinical trial.

**Clinical trial registration:**

https://classic.clinicaltrials.gov/ct2/show/NCT05759091, identifier NCT05759091.

## Introduction

Schizophrenia is a chronic and intense mental disorder that significantly affects a person’s thinking, behavior, emotions, perception of reality, and social interactions (1). Internationally, a 1% lifetime morbidity risk has been reported (2). Hallucinations, delusions, and disordered thinking are symptoms of schizophrenia that lead to impaired functioning and lifetime therapy (3). Many clients with schizophrenia respond poorly to antipsychotic treatment (4), and 4% of medication-responsive schizophrenics are hospitalized monthly (5). The span of an episode is not constant and can vary among clients, being influenced by aspects such as the episode’s characteristics and its underlying cause (6, 7).

Delusions are characterized as fixed, mistaken ideas held with confidence that are not consistent with everyday social and cultural context, appearing in around 70% of cases, most often in conjunction with hallucinations (7). Belief in a delusion can help alleviate mental and experiential tension and conflict. The degree to which a person thinks their subjective experiences correctly represent reality is delusional believability. This experience is often accompanied by an unpleasant feeling, suspicion, or a sense of strangeness (8). Previous studies have documented that the degree of believability of delusions depends on the degree of cognitive fusion. Cognitive fusion is about how we interact with our thoughts. Fusion with thoughts entails accepting them as facts, being unable to view them from various angles, and being mentally “entangled” with thoughts (9).

Psychological inflexibility has been increasingly studied as a principal factor influencing the experience of psychosis, particularly delusions. Bond, Hayes, and Barnes-Holmes (2006) define cognitive fusion as an over-abundance of attachment to the actual content of human cognition, which makes psychological flexibility that is healthy difficult or impossible (10). Psychological flexibility refers to the ability to handle negative internal experiences (such as thoughts, feelings, and recollections) in a receptive, mindful, and non-reactive way. This ability facilitates engagement in actions that align with chosen objectives and enables individuals to behave congruent with their values and goals (11, 12). In contrast, an inability to be psychologically flexible (experiential avoidance) can lead to increased sensitivity to aversive experiences and has been linked to various undesirable psychological effects, such as delusional beliefs (11, 13).

Being in contact with the present moment is defined as having a flexible awareness of experience in the here and now, encompassing sensation, emotion, cognition, and kinesthetic awareness. In line with the earlier perspective, improving psychological flexibility, mindful awareness, and cognitive defusion might enable more effective and flexible involvement in meaningful activities while lowering the believability of delusions in individuals with schizophrenia (14).

Acceptance- and mindfulness-based therapies have received much attention recently (15–17). These therapies emphasize the functional effects of internal experiences (such as thoughts, feelings, physiological sensations, and memories) more than the content and frequency of these events (16, 17). Acceptance and Commitment Therapy (ACT) illustrates one of these therapies (18). To improve psychological health, ACT employs several approaches to alter the function of internal experiences (11). Mindfulness, a crucial component of the ACT model, is the conscious awareness that arises from intentionally focusing on the present moment without judgment. Mindfulness has numerous empirically supported benefits, one of the most notable being enhancing psychological flexibility (15–18). Cognitive defusion techniques are one set of procedures utilized explicitly for this aim based on relational frame theory (RFT) (19).

Relational frame theory (RFT) is a behavior-analytic perspective on language and cognition that seeks to explain the generative nature of complex human behavior. The theory posits that individuals deduce relationships between stimuli, and their responses to stimuli are based on those relationships. This process is called derived relational responding (20). The “derived relational response” is the process of linking stimuli following a context cue that is entirely arbitrary and not dependent on the physical properties of the stimuli. Cognitive defusion alters private events’ literal meaning and behavior-regulatory function without necessarily changing their shape, frequency, or situational sensitivity; for example, “Naming your mind,” “I am having the thought that” (21). As an RFT interpretation proposes, defusion targets interfere with internal experiences to lessen their influence (18, 22). Defusion techniques are designed to interrupt fusion by decreasing the control of rules and increasing contact with direct contingencies (21).

Cognitive defusion techniques employ experiential exercises, evocative metaphors, and behavioral tasks, with logical analysis playing a minimal role (15). One crucial perspective of the cognitive defusion techniques is to minimize how beliefs dominate conscious experience and action rather than changing belief content (23). More precisely, it focuses on taking the thoughts less seriously and weakening their relationship with behavior rather than removing them entirely.

“Self-as-context” is a vital psychological concept that refers to how we perceive ourselves. It’s like looking at ourselves from a bird’s eye view, where we see all our thoughts, feelings, and experiences as part of who we are without defining us. To understand this concept better, imagine yourself as a container that holds everything about you, including your thoughts, feelings, beliefs, experiences, and more. All these are your “self-content.” Usually, we tend to equate ourselves with this self-content. For instance, if we fail at something, we might think, “I am a failure.” This is what we call an “equivalency relationship,” where we identify “I” (the self) with “failure” (a piece of self-content). However, “self-as-context” and “cognitive defusion” recommend a different approach. Instead of saying, “I am a failure,” we learn to say, “I experienced failure.” By doing so, we recognize that failure is only one of the many things in our container. It doesn’t define us but is part of our experience (24). Per the ACT paradigm, present-moment awareness is often the initial cause of cognitive defusion. Take, for instance, the “classic” ACT defusion exercise, “Leaves on the Stream.” The first step in this exercise is to practice present-moment awareness (such as breathing, body awareness, noise awareness, etc.). Then, it deliberately modifies the link between cognitive events and the ego by using the idea of thoughts as leaves floating on a stream (25).

Several studies have focused on how cognitive defusion techniques affect self-defeating thoughts (26–28). One study examined the cognitive defusion technique, “I have a thought that” (26). According to the study, when delivered defused, for example, “I have a thought that my life is meaningless,” negative self-referential statements, such as “my life is pointless,” might minimize emotional distress and boost readiness for exposure. Another study used a fast vocal repetition of a negative self-referential concept as a defusion technique (29). Indeed, the defusion technique, which involves the rapid vocal repetition of a phrase, is grounded in the principle that the context necessary for a word or phrase to retain its literal meaning can be altered through repetition. For example, the phrase “I am persecuted”. When this phrase is repeated quickly and out loud, the context that initially imbued it with its literal meaning begins to shift. As the repetition continues, the phrase’s literal meaning starts to fade, transforming it into a mere sequence of sounds (30).

According to previous studies, cognitive defusion techniques assist individuals in accepting their ideas and feelings, increasing their awareness of their environment, and improving their psychological stability (5). Thomas et al. (2014) state that ACT helps individuals with schizophrenia experience less drug-resistant psychotic symptoms, like delusions (30). That being said, no additional studies have been conducted on cognitive defusion abilities in schizophrenia. Thus, this study aimed to determine how cognitive defusion techniques affected the clients’ credibility of delusions, cognitive fusion, psychological flexibility, and mindful awareness.

## Research hypothesizes

1. Clients who participated in cognitive defusion techniques had more psychological flexibility than a waiting-list control group.
2. Clients who participated in cognitive defusion techniques had more mindful awareness than a waiting list control group.
3. Clients who participated in cognitive defusion techniques had less cognitive fusion than a waiting list control group.
4. Clients who participated in cognitive defusion techniques had less delusional believability than those in a waiting list control group.

## Methods

### Design

In contrast to a waiting list control group, this parallel-arm randomized controlled trial (RCT) looked at how well cognitive defusion strategies added to standard care TAU (treatment as usual) reduced the degree of cognitive fusion and delusion believability while increasing psychological flexibility and mindfulness awareness in schizophrenia clients. The research project was registered under the reference number NCT05759091 as a randomized clinical trial.

### Setting

A study was carried out in the inpatient psychiatric medicine wards of El-Mamoura Hospital, which is connected to the Ministry of Health and Population. The hospital can accommodate up to 948 clients. Of the 24 wards, ten are specifically designated for clients with psychosis, with five for male and five for female clients. The hospital’s 2022 statistical report indicates that these ten wards include approximately 250 to 270 clients diagnosed with schizophrenia and other psychotic disorders comorbidity.

### Sample size calculation

The participants were estimated using the G*Power Windows 3.1.9.7A program with the following parameters: effect size =0.50, α err prob =.05, Power (1-β err prob) =.80, number of groups = 2, and number of measures = 3. This study required an *a priori* sample size of seventy clients with schizophrenia, according to the calculation. Thirty-five clients comprised the control group and the intervening group’s n = 35.

### Inclusion criteria

The research study has established specific inclusion criteria. The age of the participants must fall between 18 and 60 years. They must have a diagnosis of schizophrenia but without any comorbid conditions. It is also required that they have been experiencing persistent delusions for at least six months. Literacy is a prerequisite for participation, as is the ability to communicate in a clear and relevant manner. Finally, they must express a willingness to participate in the study. The Human Rights Protection Committee of the General Secretariat of Mental Health in Egypt has recommended that the intervention be implemented solely with male patients. This recommendation is based on the inclusion of male researchers in the research team, and it aims to protect the privacy and confidentiality of female patients. The Ministry of Health and Population in Cairo has also endorsed this measure. The study’s authors followed the hospital policy and caring guidelines stipulating that female patients should receive care from female health professionals. This regulation is in place to ensure patient privacy and uphold the ethical principles governing the study setting. However, the female researchers in the current study applied the same intervention with female patients after finishing this research to ensure their rights to access care.

### Exclusion criteria

The study has established specific exclusion criteria. Clients are not eligible for the study if they have co-morbid organic brain disorders that could potentially affect cognitive functions or if they have current drug or alcohol dependence. Clients who have recently changed their antipsychotic medication or received psychoeducation or any other psychological interventions in the last month are also excluded, rather than their regular supportive psychotherapy. While the medical history of clients may document electroconvulsive therapy (ECT) sessions from previous episodes, clients who have undergone ECT at the time of data collection are not eligible.

### Random allocation

**Figure 1:**
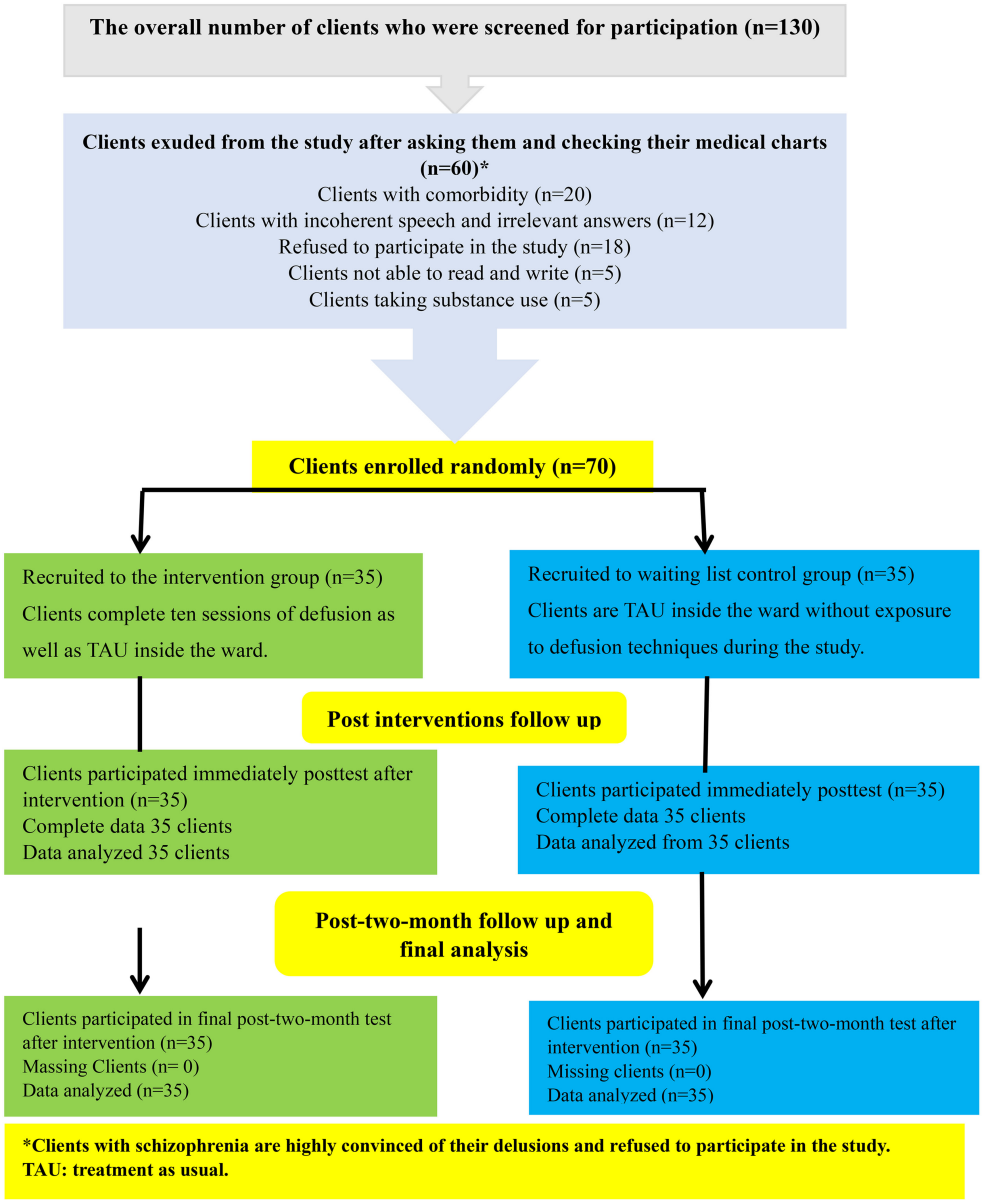
CONSORT.

The recruitment and collection of data for this study involved 130 male clients with schizophrenia. After checking their medical charts, 20 clients exhibited comorbidity, 12 demonstrated incoherent speech and irrelevant answers, five illiterate clients, five clients taking substance use, and finally, 18 clients declined to take part in the research. All these clients were excluded from the study, leaving 70 eligible male clients to meet individually (see **Figure 1**).

Participants in the trial were assigned in a 1:1 ratio to the intervention or control groups using a single-blind process. An independent researcher who did not participate in conducting the sessions generated a computer-generated allocation sequence to randomize the trial or waiting list group. The researchers maintained the single-blind procedure to prevent potential biases in the study’s results. Therefore, the participants needed to be informed about their group assignment, and the researchers who conducted the study intervention remained blind to the participant’s group allocation until the end of the study.

### Control group

A control group on the waiting list received the standard treatment (TAU) during the intervention. This treatment included the use of antipsychotics and anticholinergic medicines. The responsible psychiatrist conducted weekly supportive psychotherapy sessions for each patient, lasting approximately 30 minutes, and performed routine check-ups. Following the completion of the intervention with the active clients, this control group was subjected to the same intervention.

### Study measures

The following instruments were used to collect the study’s data

#### A sociodemographic and clinical data, structured interview schedule

The researchers developed this tool to elicit data about the client’s sociodemographic data, such as age, marital status, education level, the age of the first psychiatric consultation, duration of illness, prescribed medications, and ECT sessions.

#### Acceptance and action questionnaire-II

The self-report scale has seven items that evaluate psychological flexibility (31). The items target several vital processes: defusion, acceptance, and committed action (example: “I am afraid of not being able to control my feelings and worries”). A Likert scale is followed by each item, ranging from never true (1) to very seldom true (2), rarely true (3), occasionally true (4), frequently true (5), almost always true (6), and always true (7). The overall score, determined by adding up all of the item responses, ranges from 7 to 49, with higher scores denoting greater psychological rigidity. Cronbach’s alpha of.93 has been found in earlier studies (32). Using Cronbach’s α=0.75, the conventional Arabic version of the scale demonstrated good internal consistency.

#### The cognitive fusion questionnaire-7

Gillanders et al. (2014) created the CFQ-7 to measure a person’s degree of cognitive fusion (e.g., “My thoughts cause me distress or emotional pain”). Using a 7-point Likert scale that goes from 1 (never true) to 7 (always true), respondents score each of the seven items (33). Higher CFQ scores indicate greater cognitive fusion; the total score can vary from 7 to 49. Research has demonstrated that the scale exhibits strong internal consistency, as evidenced by Cronbach’s α=0.93 (34, 35). The internal consistency of the scale with Cronbach’s α was 0.88 in the current investigation. The researchers performed an exploratory factor analysis to determine whether the Arabic-translated tool was valid. Bartlett’s sphericity test results were statistically significant (P = 0.000), and the Kaiser-Meyer-Olkin sampling adequacy was 0.87, suggesting that the data were well suited for factor analysis. After rotation, the loadings changed from 0.62 to 0.83 to 0.71 to 0.84. The difference explained 65.49% of the variance.

#### Southampton mindfulness questionnaire

The SMQ is a 16-item questionnaire developed by Chadwick et al. (2008) to measure how mindfully people react to stressful thoughts, images, and sensations that are significant psychopathological features in various clinical illnesses (36). A 7-point Likert scale, ranging from “strongly disagree” (0) to “strongly agree” (6), was used to grade the replies. The total score range was 0-96. Eight phrases are presented positively, and eight adversely. It was constructed to assist the study process and results of mindfulness for psychosis. It comprises four constructions that form a predisposition toward mindfulness in daily life. These elements include (1) non-aversion vs. experiencing avoidance and (2) decentered awareness of cognitions versus being absorbed in reacting to them. (3) letting one’s challenging ideas pass without ruminating on or worrying about them; (4) accepting and not judging one’s unpleasant thoughts. The scale displayed acceptable reliability with a Cronbach’s alpha of 0.89 (36). Our study confirmed the internal consistency with Cronbach’s α =0.82. An exploratory factor analysis was conducted to examine the validity of the measure used in the study. The analysis showed that the data were highly suitable for factor analysis, as indicated by Kaiser-Meyer-Olkin’s sampling adequacy of 0.88 and Bartlett’s statistically significant sphericity test (P = 0.000). The loadings before rotation ranged from 0.66 to 0.80, and after rotation, they ranged from 0.61 to 0.85, accounting for 66.52% of the variation.

#### Psychotic symptom rating scales (PSYRATS-D)

The PSYRATS instrument is a self-reported questionnaire commonly used to assess auditory hallucinations and delusions in clinical and research settings (37). Only the Delusion subscale (PSYRATS-D), which consists of six questions intended to gauge various features of delusions, was employed in this research. Four items on the first PSYRATS-D subscale, which focuses on cognitive interpretation, gauge the degree and length of obsession with delusions, conviction, and disruption of everyday life due to beliefs. Two items on the second subscale, which gauges emotional distress, rate the degree and type of suffering brought on by delusions. Each item on the PSYRATS-D subscale was rated by participants on a five-point Likert scale, with 0 representing no endorsement and 4 representing full endorsement. Higher scores on the PSYRATS-D indicate more severe delusions. The total score range for the test is 0 to 24. High test-retest reliability (r = 0.70) and inter-rater reliability (0.99–1) have been reported for the PSYRATS-D (38). A Cronbach’s α of 0.85 indicated dependability in the current investigation.

### Procedure

#### Preparation and planning phases

The researcher developed a structured interview schedule for sociodemographic and clinical data. The AAQII standard Arabic version was applied. Arabic and back translations were performed for the CFQ-7, SMQ, and PSYRATS-D. Seven psychiatric nursing specialists served as the jury that evaluated the scales’ face validity. The scales’ Arabic translations were assessed for content translation, clarity, and relevancy. The instruments’ Lawshe Content Validity Ratio of 1 indicates that the information within them is legitimate. Compared to 0.99, this figure was a more significant outcome.

#### Preparation and implementation of defusion skills

A structured interview schedule was conducted in a private, comfortable room at three different time points: the beginning of the intervention (baseline), the end of the intervention (endpoint), and months after follow-up, using the study tools AAQII, CFQ-7, SMQ, and PSYRATS-D. Each interview lasted from 20 to 25 minutes, depending on the client’s attention, concentration, and level of understanding.

One of the researchers completed an 8-week online academic training course in Acceptance and Commitment Therapy (ACT) to obtain a 16-credit credential from Harris (39). The researchers used an Arabic protocol for applying ACT to schizophrenia clients tailored to Egyptian culture (40). In addition to the principles and applications of ACT with clients with psychosis (5, 18, 41), the researchers designed a psycho-educational module of defusion skills from ACT. The intervention aimed to help clients with delusions develop skills to cope with their experiences.

The intervention consisted of ten 60-minute sessions that focused on mindfulness exercises, cognitive defusion techniques, and other skills such as Silly Voice, Naming the Story, Passengers on the Bus, Saying it Slowly, Leaves on a Stream, Taking Your Mind for a Walk, and Carrying Cards (**Table 1**). Each session’s development focused on both broad and detailed goals. The defusing skills were taught using imagery, metaphors, role-plays, and simulations; corrective feedback and homework images were also included.

**Table 1:** Cognitive-defusion techniques sessions for clients with schizophrenia.

Sessions	Interventions
**Session 1** Prepare the clients to be willing to use defusion skills instead of avoidance.	▪ Evaluate and thoroughly comprehend the delusion experience, including a fundamental understanding of the delusion phenomena. ▪ The researcher encouraged the client to investigate his typical emotional control strategies to deal with delusions and their impact on his life. ▪ Face the client with the standard emotional control agenda employed in reaction to the delusions (confronting agenda). ▪ The selection points model was modified to illustrate the relationship between sources of fusion and delusional beliefs and how these factors affect deviating from the life the client desires to live. It also conveys the importance of acceptance and creating hopelessness from previous clients’ coping strategies.
**Session 2** Start applying the acceptance and defusion skills.	▪ Tug of war with a monster metaphor and Quicksand metaphor were used to validate willingness by dropping the robe massage. ▪ Defusion and acceptance go hand in hand because both aim to lessen the effects of dysfunctional rule-governance so that people can achieve their objectives. The Swamp Metaphor is a prevalent acceptance metaphor. ▪ Assign the homework.
**Session 3** Defusion by being Present: How to Feel and Be Here and Now	▪ The clients were taught to focus on their surroundings rather than their inner experiences (thoughts, feelings, and bodily sensations). ▪ The researchers encourage the client to let go of the fighting thoughts and instead focus on the present moment. ▪ The researchers were practicing the dropping anchor exercise
**Session 4** Defusion techniques	▪ When referring to behavior that altered due to internal experiences of any thoughts and feelings that surfaced during the session, the researchers advised the client to use the language of “noting,” such as stating, “I’m noticing that…” ▪ The client was encouraged to see a movie depicting the Metaphor River of Thoughts as delusional ideas and sensations came and went from the viewpoint of an observer (Mindfulness exercises). ▪ Assign the homework.
**Session 5** Defusing from the thought: Living with Thoughts and Delusional beliefs	▪ By having the client practice distancing exercises to detach himself from the conceived self of the delusion experience, the researcher helped the client reduce the effect of the delusion content on his actions. ▪ Milk. Milk. Milk exercise (repeated delusion content more than 30 times) aims to expose the client to a stimulus’s direct qualities, which may initially be less noticeable than its derived functions. ▪ The Cards Game and Swamp Metaphor: The Cards Game clarifies that covert actions and private happenings are distinct. In this practice, clients are instructed to jot down a few unsettling ideas on index cards. The client was instructed to walk around the room while holding a folded paper. The client opened the paper and read “Content of Delusion” after going around the room. The client was asked to consider if they could carry out a job despite the content of one of their uncomfortable thoughts when presented. The client learns through this activity that personal actions and events are merely temporally connected, not causally. ▪ Homework assignment.
**Session 6** Defusing from the thought: Living with Thoughts and Delusional beliefs	▪ “I am a prophet or a significant man, etc.”. We occasionally react to our ideas as if they presented the real world. This exercise helps the client remember that a thought is just that—a thought—and may not correspond to what will occur. Encourage the client to put the technique to use and track any behavioral changes they see across all defusion activities. ▪ To support the message of minimizing delusional material, the researcher and the client performed “Take your mind for a Walk” in the hospital garden. ▪ Homework assignment.
**Session 7** Defusing from the thought: Living with Thoughts and Delusional beliefs	▪ Using the metaphor Passenger on the bus: It is necessary to establish analogies between any patient’s answers for the bothersome passengers’ presence and any daily strategies to combat their delusions or other thoughts. The similarities between the client’s delusions and the furious bus passengers should be highlighted. The analogy emphasizes how crucial it is to maintain control of the bus and lead it toward things that matter to you, even while other passengers annoy the driver (even with delusions or other bizarre ideas). The client should be asked what they can do after explaining this metaphor. People typically respond symbolically by offering remedies from their own experiences. The therapist must attempt to get the client to consider the outcomes of the method they employed in practice and if it was successful in “throwing the passengers off the bus” at this point. ▪ “Selling your thoughts exercise. ▪ Homework assignment.
**Session 8** Defusion by creating distance between the client and his mind and differences between self as content, self as context, and observing self.	▪ The researchers observed delusional ideas and negative thoughts on several occasions to assist the client in comprehending the various aspects of self-conceptualization. ▪ The researcher highlighted to the client the contrast between the self who sees the delusional ideas or negative thoughts that develop and the self who experiences such thoughts or delusions. ▪ “Taking your mind for a walk. “ This metaphor is a dramatization exercise in which the client is separated from his or her mind. This practice is meant to help the client learn to behave without the assistance of his or her thoughts. In this regard, it is essential to note that the exercise includes assessing how much the client’s delusions control them. ▪ Assign the homework.
**Session 9** Defusion by creating distance between the client and his mind and differences between self as content, self as context, and observing self.	▪ The “Constant You” ▪ The researcher utilized a movie to symbolically represent the leaves on the stream as the client’s ideas, feelings, and delusional beliefs, asking them to see them as thoughts, feelings, and drifting leaves in a stream without trying to stop or control them. ▪ The researcher formed and highlighted the difference between the self that evaluates and the outputs and components of self-evaluations. Playing chess with the client, the researcher utilized a chessboard metaphor and chess pieces to show the difference between ideas and self “Chessboard metaphor.” ▪ Assign the homework.
**Session 10** Ending the Therapy	▪ The researcher summarized the defusion techniques. ▪ Cleared any misconception and emphasized the importance of repeating the learned defusion skills.

#### Evaluation of the effectiveness of cognitive defusion skills

The last phase, “the posttest,” is done twice: immediately after the end of all sessions and a two-month follow-up utilizing tools AAQII, CFQ-7, SMQ, and PSYRATS-D. Data was gathered over 12 months, including an active session starting in September 2022 and ending in August 2023. A follow-up was then conducted two months later.

#### Data analysis

The IBM SPSS software package, version 26.0, was used to evaluate the data gathered. The data, including the minimum and maximum, averages, standard deviations, numbers, and percentages, are described using descriptive statistics. The Cronbach’s Alpha test was used to evaluate the study instruments’ dependability. Factor analysis was used to verify the validity of the translated instruments (CFQ-7, SMQ, and PSYRATS-D). The Shapiro-Wilk test was used to determine if the variable distribution was normally distributed. Because of the small sample size, the Chi-square test using Monte Carlo simulation was utilized for categorical variables. For quantitative data that were regularly distributed, a student’s t-test compared two categories. The Mann-Whitney U test compared two groups of quantitative variables with non-normal distributions. The changes over time were analyzed using an ANOVA with repeated measurements, and for multiple comparisons between the three periods in each group, a Bonferroni adjustment was applied. A 5% and 1% statistical significance threshold was used.

## Results

**Table 2** reveals that the clients in the intervention group were, on average, 31.34 (4.67) years old, whereas the control group’s average age was 32.29 (6.35). Most clients were single (71.4% for the intervention group and 60.0% for the control group), and more than half (51.4%) had a college degree. With a mean age of 26.43 (2.97) in the intervention group and 25.80 (2.77) in the control group, the majority of the clients were unemployed (40.0% for the intervention group and 60.0% for the control group). They were diagnosed with schizophrenia between the ages of 25 and 30. Furthermore, the similarity between the two groups was shown by the lack of a statistically significant difference in the sociodemographic features between the intervention and control groups.

**Table 2:** Distribution of the studied clients with schizophrenia regarding their sociodemographic and clinical data characteristics.

Socio-demographic and clinical characteristics	Study group (n=35)		Control group (n=35)		*Test of sig.*	*P*
	N	%	N	%		
Age of the clients (years)						
20-	2	5.7%	2	5.7%	**χ^2^** = 0.757	^MC^p= 0.839
25-	9	25.7%	12	34.3%		
30-35	24	68.6%	21	60.0%		
**Min-Max**	24-41		23-45		t = 0.707	0.482
**M (SD)**	31.34 (4.67)		32.29 (6.35)			
Educational level						
Basic education	18	51.4%	18	51.4%	**χ^2^** = 0.257	^MC^p= 1.000
Secondary education	14	40.0%	13	37.1%		
Higher education	3	8.6%	4	11.4%		
Marital status						
Single	25	71.4%	21	60.0%	**χ^2^** = 2.528	^MC^p= 0.435
Married	8	22.9%	8	22.9%		
Divorced	2	5.7%	5	14.3%		
Occupation						
Unemployed	14	40.0%	21	60.0%	**χ^2^** = 4.118	^MC^p= 0.397
Student	1	2.9%	2	5.7%		
Employee	7	20.0%	3	8.6%		
Craft worker	10	28.6%	7	20.0%		
Trade worker	3	8.6%	2	5.7%		
Age of disease						
20-	11	31.4%	12	34.3%	0.603	^MC^p= 0.881
25-	19	54.3%	20	57.1%		
30-35	5	14.3%	3	8.6%		
**Min-Max**	22.0-35.0		22.0-35.0		t = 0.915	0.364
**M (SD)**	26.43(2.97)		25.80 (2.77)			
Type of medications						
Atypical antipsychotics	13	37.1%	7	20.0%	**χ^2^** = 2.520	0.112
Typical antipsychotics	11	31.4%	19	54.3%	**χ^2^** = 3.733	0.053
Mixed typical and atypical	11	31.4%	9	25.7%	**χ^2^** = 0.280	0.597
Mood stabilizers	5	14.3%	7	20.0%	**χ^2^** = 0.402	0.526
Anticholinergics	20	57.1%	18	51.4%	**χ^2^** = 0.230	0.631
Previous Electroconvulsive therapy sessions						
Yes	11	31.4%	9	25.7%	**χ^2^** = 0.280	0.597
No	24	68.6%	26	74.3%		

χ^2^, Pearson Chi-square test; MC, Monte Carlo.

*no significant difference if P > 0.05.

The psychological inflexibility mean scores and standard deviations (SD) for the intervention and control groups are shown in **Table 3** at three different time intervals: baseline, immediately after the intervention, and two months later. The results of the inter-group analysis demonstrate that the mean scores for the intervention group decreased, with a large effect size of 84.6%, from 32.91 SD (6.03) at baseline to 23.06 SD (6.22) and 26.83 SD (3.49) post-two month. This decrease was statistically significant. Immediately following the intervention and at the two-month follow-up, the control group’s mean score of psychological inflexibility increased compared to the baseline.

**Table 3:** Description of mean scores and standard deviations of AAQ-II for the study and control groups at baseline, immediate post, and post two-month.

Scale		Study group (n= 35)		Control group (n= 35)		*t*	*p*
		M	SD	M	SD		
**AAQ.II**	**Baseline**	32.91	*6.03*	*31.60*	*7.95*	*0.779*	*0.439*
	**Post**	23.06	6.22	32.14	8.71	5.020^**^	<0.001^**^
	***Post-two-month***	26.83	3.49	33.83	9.42	4.124^**^	<0.001^**^
	**F**	71.185^**^		2.331			
	**P**	<0.001^**^		0.105			
	**η^2^**	0.846		0.124			

AAQ-II, Acceptance and Action Questionnaire-II; t, independent t-test; F, ANONA with repeated measures; η^2^, Partial Eta Squire.

** Statistically significant p-value at ≤.001.

The mean scores and standard deviations for cognitive fusion in the intervention and control groups’ baselines two and immediately following the intervention are displayed in **Table 4** as an example of the intergroup analysis. The table shows that, with a large effect size of 82.1%, the mean scores of cognitive fusions for the intervention group decreased statistically significantly from 34.37 SD (5.62) to 23.40 SD (4.75) immediately following the intervention and 24.63 SD (4.58) months later. On the other hand, the mean score of cognitive fusion increased immediately following the intervention and two months later in the control group.

**Table 4:** Description of mean scores and standard deviations of CFQ-7 for the study and control groups at baseline, immediate post, and post two-month.

Scale		Study group (n= 35)		Control group (n= 35)		*t*	*p*
		M	SD	M	SD		
**CFQ.7**	**Baseline**	34.37	5.62	34.94	6.16	0.405	0.686
	**Post**	23.40	4.75	35.91	6.64	9.067^**^	<0.001^**^
	***Post- two -month***	24.63	4.58	38.34	6.02	10.719^**^	<0.001^**^
	**F**	126.233^**^		5.712^*^			
	**P**	<0.001^**^		0.005^*^			
	**η^2^**	0.821		0.207			

CFQ-7, Cognitive Fusion Questionnaire-7; t, independent t-test; F, ANONA with repeated measures; η^2^, Partial Eta Squire.

* statistically significant p-value at ≤.05.

** Statistically significant p-value at ≤.001.

The inter-group analysis of the mindfulness skills means scores and standard deviations (SD) for the intervention and control groups at three different time points—baseline, right after the intervention, and post-two months after the intervention—is shown in **Table 5**. The mean scores of the intervention group increased statistically significantly between baselines and immediately after the intervention, from 59.29 SD (3.30) to 67.43 SD (4.70) and 74.80 SD (4.44) post-two months, with a large effect size of 86.9%, as shown in the table. By comparison, the control group’s mean score for mindfulness abilities significantly decreased two months after and immediately after the intervention.

**Table 5:** Description of mean scores and standard deviations of SMQ for the study and control groups at baseline, immediate post, and post two-month.

SMQ		Study group (n= 35)		Control group (n= 35)		*t*	*p*
		MSD		M	SD		
** SMQ**	** Baseline**	59.29	3.30	58.57	2.66	0.996	0.323
	**Post**	67.43	4.70	62.57	7.42	3.270^*^	0.002^*^
	***Post- two -month***	74.80	4.44	63.57	8.90	6.680^**^	<0.001^**^
	**F**	8.90		8.90			
	**P**	8.90		8.90			
	**η^2^**	8.90		8.90			

SMQ, Southampton Mindfulness Questionnaire; t, independent t-test, F ANONA with repeated measures; η^2^, Partial Eta Squire.

* Statistically significant p-value at ≤.05.

** Statistically significant p-value at ≤.001.

The PSYRATS-D mean scores and standard deviations (SD) for the intervention and control groups at three different time intervals are shown in **Table 6**: baseline, just after the intervention, and post-intervention, two months later. The total mean scores of the cognitive interpretation subscale and its items for the intervention group decreased from 10.26 SD (1.72) to 8.11 SD (1.11) immediately after the intervention and to 8.80 SD (1.57) two months later, a highly statistically significant decline that is displayed in the table. In contrast, the control group’s mean score rose during the three periods. Concerning the emotional qualities of delusion, the table shows that the total mean scores for the intervention group decreased from 15.34 SD (3.06) to 12.14 SD (2.29) immediately after post-intervention and to 13.06 SD (3.56) post-two months. This decline in mean scores was highly statistically significant. In contrast, the control group’s mean score rose during the three periods. Additionally, the data demonstrates a highly statistically significant drop in the intervention group’s mean score of delusional beliefs, with a moderate effect size of 66%, from 15.34 SD (3.06) to 12.14 SD (2.29) immediately following the intervention and 13.06 SD (3.56) after two months.

**Table 6:** The PSYRATS-D means scores and SD at baseline, immediate, post, and post-two-month time points for the study and control groups.

PSYRATS-D		Study group (n= 35)						Control group (n= 35)						*t(p)*		
		*Baseline*		*Post*		*Post-two months*		*Baseline*		*Post*		*Post-two months*				
		*M*	*SD*	*M*	*SD*	*M*	*SD*	*M*	*SD*	*M*	*SD*	*M*	*SD*	*Baseline*	*Post*	*Post-two months*
**Cognitive Interpretation**	**Amount of preoccupation**	2.57	0.74	2.09	0.61	2.09	0.78	2.69	0.63	2.74	0.56	2.66	0.59	0.696 (0.489)	4.683^**^ (<0.001^**^)	3.451^**^ (0.001^**^)
	**Duration of preoccupation**	2.57	0.56	2.09	0.66	2.03	0.51	2.49	0.70	2.71	0.79	2.63	0.73	0.566 (0.573)	3.620^**^ (0.001^**^)	3.973^**^ (<0.001^**^)
	**Conviction**	2.91	0.78	2.23	0.65	3.09	0.95	2.91	0.66	3.31	0.68	3.11	0.72	0.0 (1.000)	6.871^**^ (<0.001^**^)	0.142 (0.888)
	**Disruption of life caused by beliefs**	2.20	0.58	1.71	0.62	1.60	0.50	2.46	0.61	3.03	0.79	3.0	0.77	1.800 (0.076)	7.762^**^ (<0.001^**^)	9.062^**^ (<0.001^**^)
**Total score subscale**		10.26	1.72	8.11	1.11	8.80	1.57	10.54	1.60	11.80	1.76	11.40	1.79	0.720 (0.474)	10.482^**^ (<0.001^**^)	6.473^**^ (<0.001^**^)
**F**		27.896^**^						8.430^**^								
**p**		<0.001^**^						0.001^**^								
**η^2^**		0.623						0.387								
**Emotional Characteristics**	**Amount of Distress**	2.63	0.77	2.11	0.96	2.14	1.40	2.57	0.88	2.80	1.08	2.91	0.70	0.288 (0.774)	2.804^*^ (0.007^*^)	2.920^*^ (0.005^*^)
	**Intensity of Distress**	2.46	0.95	1.91	0.89	2.11	0.93	2.46	0.85	2.77	0.94	2.89	0.63	0.0 (1.000)	3.919^*^ (<0.001^*^)	4.054^*^ (<0.001^*^)
**Total score subscale**		5.09	1.54	4.03	1.58	4.26	2.15	5.03	1.50	5.57	1.77	5.80	0.87	0.157 (0.876)	3.846^*^ (<0.001^*^)	3.942^*^ (<0.001^*^)
**F**		6.881^*^						6.262*								
**p**		0.002^*^						0.003^*^								
**η^2^**		0.460						0.248								
**Total score**		15.34	3.06	12.14	2.29	13.06	3.56	15.57	2.73	17.37	2.89	17.20	2.23	0.330 (0.742)	8.387^**^ (<0.001^**^)	5.837^**^ (<0.001^**^)
**F**		20.174^**^						13.532^**^								
**p**		<0.001^**^						<0.001^**^								
**η^2^**		0.660						0.432								

PSYRATS-D, Psychotic Symptom Rating Scales–Delusion; t, independent t-test; F, ANONA with repeated measures; η^2^, Partial Eta Squire.

* Statistically significant p-value at ≤.05.

** Statistically significant p-value at ≤.001.

## Discussion

A diverse range of complex mental disorders, including positive, negative, and emotional symptoms as well as cognitive dysfunctions, are exhibited by individuals with schizophrenia spectrum disorders (SSD) (42). Thus, there has been a growing interest in the latest developments in third-wave cognitive-behavioral therapies (CBT) to enhance clients’ relationships and attitudes toward themselves and their symptoms. Third-wave cognitive behavioral therapy (CBT) focuses on how a person interacts with and responds to experiences and symptoms rather than trying to change them.

These techniques are predicated on concepts such as cognitive defusion, accepting oneself with compassion, and nonjudgmental awareness (25). The purpose of the current study was to look at how cognitive defusion procedures affected the plausibility of delusions, psychological flexibility, mindful awareness, and cognitive fusion (CF) in schizophrenia clients.

Initially, it was assumed that clients using cognitive defusion techniques were more psychologically flexible than those in the control group. The current study results showed a considerable positive effect of the cognitive defusion (CD) technique on psychological inflexibility among individuals with schizophrenia, which is consistent with our initial hypothesis. These findings could be explained by the idea that removing oneself from the direct impact of personal events can help people accept them, relate to their values and environment, and focus their behavior on the committed activities that are currently available, thus improving psychological flexibility. This outcome demonstrates how well cognitive defusion treatments work to achieve their therapeutic objectives in schizophrenia clients.

Furthermore, it has been noted that the detrimental effects of cognitive fusion make it difficult for clients to act in the present in a way consistent with their values, ultimately compromising their psychological flexibility (43, 44). Furthermore, several research studies have documented the noteworthy impact of cognitive diffusion on the psychological adaptability of individuals diagnosed with schizophrenia (45–47).

This study also looked at the second hypothesis: clients who used cognitive defusion techniques were more mindful than those in the control group. In this regard, the current study demonstrated that the intervention group’s gain in mindfulness was far more significant. The tight relationship between mindfulness and ACT and defusion dimensions, such as acceptance, present moment awareness, and self-as-context, may help to explain this conclusion. Our results also demonstrate that psychological flexibility requires mindfulness. Böge et al. (2022) found that those with lower cognitive fusion scores were likelier to exhibit greater psychological flexibility and mindfulness (46). Furthermore, additional research has demonstrated the vital role that cognitive defusion plays in enhancing mindfulness in schizophrenia clients (48–50).

The third hypothesis, which concludes, concerns how the CD technique affects cognitive fusion and delusional belief in the schizophrenia clients under study. Cognitive fusion is when people lose awareness of their thoughts and confuse them with reality. The current findings demonstrated the effectiveness of cognitive defusion in reducing cognitive fusion and delusion believability in schizophrenia clients under study. This result could be explained by the effectiveness of CD in helping clients accept their unpleasant feelings and beliefs (51). Psychological flexibility is diminished, and psychological issues arise in people with pathological cognitive fusion and experiential avoidance (25).

Consistent with this, Masuda et al. (2010) documented the superiority of the defusion technique in lowering thought believability and negative self-referential thoughts in the group under study (27). Furthermore, Blackledge (2007) proposed that defusion activities unsettle the literal context. This effect results from changes to the contextual elements that give thoughts their meaning through the change of functions (22). Assaz et al. (2018) demonstrated that thinking is not a compelling story reflecting reality by elucidating the impact of cognitive defusion activities on thought fusion and believability (52, 53).

The cognitive defusion technique also effectively reduced the amount and intensity of emotional distress. These could be explained by the fact that cognitive diffusion techniques aid individuals in enhancing their cognitive flexibility, particularly when their thoughts hinder their enjoyment of life or alignment with their values. It encourages individuals to disengage from their thoughts instead of accepting them as irrefutable facts. This disengagement is facilitated by redirecting focus from the substance of thoughts to the act of thinking itself. The outcome is a reduction in mental distress and rumination, leading to a more equilibrated viewpoint. These results align with Masuda et al. (2010), who posted that cognitive defusion decreased the stimulus effects as emotional discomfort, which is strongly related to these thoughts more so than comparison conditions across all participants. Identifying the fundamental mechanism underlying the effects of cognitive defusion exercises is challenging, and research on this subject still needs to be conducted (27).

### Strength and limitations

This study represents the first exploration of the effectiveness of cognitive defusion methods in managing cognitive fusion (CF) and the plausibility of delusions in individuals with schizophrenia. It builds upon the work of El Ashry et al. (2021), who examined the use of acceptance and commitment therapy (ACT) sessions to manage persistent auditory hallucinations within the Egyptian cultural context (40). The current study extended this approach to address delusions, a common symptom of psychosis in clients with schizophrenia, using cognitive defusion techniques. The results indicated a promising reduction in the severity of delusions. However, the study faced several challenges. Some clients were hesitant to participate due to the nature of their delusional beliefs and their high level of conviction. The study’s sample was exclusively male, limiting the findings’ generalizability, although it decreased the gender confounding factor. Future research should expand the demographics to include factors such as race/ethnicity, socioeconomic status, and sexual orientation. As well as including female participants must be considered in future researches. The short follow-up period in the current study raises questions about the long-term effects of the intervention, suggesting the need for future studies with more extended follow-up periods. Additionally, the effects of the intervention on individuals with schizophrenia who also have comorbid conditions such as Obsessive-compulsive symptoms, Depressive symptoms could be explored in future research. Lastly, examining the intervention’s effectiveness in different cultural contexts could provide insights into its applicability across various cultures.

## Conclusion

Cognitive defusion skills contribute positively to clients with schizophrenia who suffer from persistent delusional beliefs. The intensity of delusion and cognitive fusion was positively diminished. Both mindfulness skills and psychological flexibility improved.

### Implications for practice

Recently, there has been a lot of interest in therapies focused on acceptance and mindfulness. Based on relational frame theory, cognitive defusion is one set of procedures explicitly used for this goal. The impact of cognitive defusion strategies on cognitive fusion (CF) and the plausibility of delusions among schizophrenia clients is being examined for the first time in this study. According to this study, those who have schizophrenia should employ cognitive defusion strategies. These abilities would facilitate adaptable delusion control tactics for cognitive and emotional delusion. Consequently, psychiatric mental health nurses need to be trained in “cognitive defusion skills” to assist clients with schizophrenia in becoming aware of their emotions and changing their coping mechanisms for delusional beliefs.

## Data availability statement

The raw data supporting the conclusions of this article will be made available by the authors, without undue reservation.

## Ethics statement

All methods were carried out according to the relevant guidelines and regulations of the Declaration of Helsinki (DoH-Oct2008). The Ethical Committee of Alexandria University's Faculty of Nursing (IRB00013620/63/9/2022) reviewed, accepted, and approved verbal informed consent for the study proposal. An agreement from the head of the psychiatric outpatient clinics at the Main Alexandria University Hospital for Psychiatric Medicine and the Clients' Rights Protection Committee of the Ministry of Health and Population's General Secretariat of Mental Health in Cairo (code. 986/2022) was obtained to conduct the trial. Every participant in the therapy was briefed about the objectives of the therapy and their written informed consent was obtained for the publication of any potentially identifiable images or data included in this article. Alongside the consent given by the client, written consent was also obtained from their guardians. The confidentiality of the client's name and personal information served to safeguard it. It was emphasized that the client is free to refuse or stop participating in the study at any time.

## Author contributions

AE-A: Writing – original draft, Investigation, Data curation, Conceptualization. EA: Writing – original draft, Methodology. ST: Writing – original draft, Formal analysis, Data curation. MK: Writing – original draft, Supervision, Software, Project administration, Investigation. FA: Writing – original draft, Conceptualization. AA: Resources, Writing – review & editing, Funding acquisition. SF: Writing – review & editing, Project administration, Funding acquisition. ME-S: Writing – original draft, Methodology, Data curation, Conceptualization.
